# Clinical Psychological Correlates of Malocclusion: Body Image Concerns and Oral Health-Related Quality of Life

**DOI:** 10.3390/jcm15186965

**Published:** 2026-09-08

**Authors:** Emanuele Maria Merlo, Federica Sicari, Giulia Gentile, Liam Alexander MacKenzie Myles, Riccardo Nucera, Marco Portelli, Angela Militi

**Affiliations:** 1 Department of Biomedical, Dental Science and Morphological and Functional Images, University of Messina, 98100 Messina, Italy; giulia97gentile@libero.it (G.G.); riccardo.nucera@unime.it (R.N.); marco.portelli@unime.it (M.P.); angela.militi@unime.it (A.M.); 2 Department of Cognitive Sciences, Psychology, Educational and Cultural Studies (COSPECS), University of Messina, 98100 Messina, Italy; federicasicari7@gmail.com; 3 Berkshire Healthcare NHS Foundation Trust, Bracknell RG12 2UT, UK; liam.a.myles@outlook.com

**Keywords:** body image, clinical psychology, malocclusion, oral health-related quality of life, orthodontics

## Abstract

**Background**: Malocclusion is a highly prevalent condition, affecting approximately 56% of the global population. Beyond its functional and physical characteristics, malocclusion has been associated with psychosocial outcomes, particularly body image concerns and perceived quality of life. The present study aimed to investigate the associations among Angle class, body image concerns, and oral health-related quality of life. **Methods**: A total of 136 adolescents aged 10 to 19 years (M = 15.49; SD = 2.64; 47.8% males and 52.2% females) were recruited from the “Gaetano Martino” University Hospital of the University of Messina. Following the clinical diagnosis and classification of malocclusion according to Angle’s classification (Class I, Class II, and Class III), participants underwent a psychodiagnostic assessment comprising a sociodemographic questionnaire, the Italian version of the Body Image Concern Inventory (I-BICI), and the Oral Health Impact Profile (OHIP-14). **Results**: Greater body image concerns were significantly associated with poorer oral health-related quality of life. Female participants reported significantly higher levels of body image concerns than males, whereas no significant sex differences emerged in OHIP-14 scores. Generalized linear models further showed that Angle class was significantly associated with body image concerns and oral health-related quality of life after controlling for age and sex. **Conclusions**: The findings indicate that Angle class is associated with body image concerns and oral health-related quality of life in adolescents with malocclusion. These results highlight the relevance of considering body image concerns and patient-reported oral health-related quality of life alongside conventional orthodontic assessment, supporting a more comprehensive, patient-centered approach to orthodontic care.

## 1. Introduction

Malocclusion is widely recognized as a condition associated with significant psychological and psychosocial challenges [1,2,3,4]. Recent epidemiological evidence has estimated its global prevalence at approximately 56% of the general population [5], underscoring its substantial public health burden. Over the past decade, increasing scientific attention has focused on the relationship between physical health conditions and psychological functioning, highlighting the complex interplay between physical alterations and mental well-being [6,7,8,9,10,11].

The psychological burden associated with malocclusion appears to be particularly pronounced during adolescence, a developmental stage characterized by heightened sensitivity to appearance-related concerns and interpersonal evaluation. Within this context, body image represents a central psychological construct. Body image has been defined as the explicit and conscious representation individuals develop of their body size, shape, and physical characteristics [12]. According to Schilder [13], body image reflects the mental representation of the body, emphasizing the interaction among psychological, neurological, and sociocultural dimensions involved in bodily experience. Accordingly, body image encompasses the subjective experience of the body and reflects the dynamic interplay between cognitive and emotional processes, shaped by personal experiences and social interactions [14,15].

Current literature suggests that body image is profoundly influenced by affective experiences, interpersonal relationships, sociocultural standards, and individual developmental history [16,17,18,19,20,21,22]. Consequently, disturbances affecting facial appearance and dental structure may exert a substantial influence on psychological functioning and self-perception. In this context, facial dysmorphia and appearance-related concerns have frequently been reported among individuals with malocclusion [23,24,25].

Alterations involving dental and craniofacial structures may therefore represent critical determinants of patients’ subjective experiences and psychosocial adjustment [26,27]. Previous evidence has shown that discomfort, functional limitations, and perceived disability associated with oral conditions may contribute substantially to the development and persistence of psychological distress [28,29]. More broadly, the literature consistently indicates that chronic physical and appearance-related difficulties may adversely affect emotional well-being, social functioning, and overall mental health [2,30]. Within this context, several authors have emphasized the importance of integrating psychological support into the management of patients with oral and craniofacial conditions [31].

The existing literature has extensively investigated variables such as self-esteem [32,33], quality of life [34], psychosocial functioning associated with craniofacial conditions [35], and the social implications of oral and facial alterations [10,36]. Nevertheless, most available studies have primarily focused on the outcomes of orthodontic and dental interventions [4,31,37], whereas comparatively less attention has been devoted to the psychological dimensions underlying patients’ experiences. Consequently, further investigation of the psychological correlates of oral health conditions is warranted to establish a stronger theoretical and clinical foundation for integrated multidisciplinary interventions.

Improvements in oral and craniofacial conditions are frequently associated with enhanced psychological well-being and quality of life, suggesting that the benefits of treatment extend beyond purely functional and sensorimotor domains [38]. Recent studies have increasingly highlighted the psychosocial impact of malocclusion and related dental conditions, underscoring the need to further clarify the relationship between oral health difficulties and psychological functioning [3,39,40,41].

The present study aimed to investigate the associations among occlusal status, body image concerns, and oral health-related quality of life in adolescents with malocclusion.

### Hypotheses

**Hypothesis 1.**  *Significant associations would emerge between body image concerns and oral health-related quality of life*.

**Hypothesis 2.**  *Male and female participants would significantly differ in body image concerns and oral health-related quality of life*.

**Hypothesis 3.**  *Age, sex, and Angle class would be independently associated with body image concerns and oral health-related quality of life*.

## 2. Methods

### 2.1. Participants and Inclusion Criteria

This study included consecutive patients attending the Orthodontic Clinic of the “Gaetano Martino” University Hospital, University of Messina, as part of routine clinical practice. All participants had a diagnosis of malocclusion and were independently examined by two orthodontists to determine their occlusal status according to Angle’s classification of dental malocclusion (Class I, Class II, and Class III) [42]. Any disagreements between the two orthodontists were resolved through discussion and consensus, and the agreed classification was used for the analysis. No formal pre-study examiner calibration was performed, and no quantitative measure of inter-examiner agreement was obtained. Angle class was considered a categorical clinical classification of dental sagittal relationships and was not interpreted as an ordinal measure of malocclusion severity. The inclusion criteria were as follows: absence of proximal caries or restorations, absence of craniofacial anomalies, absence of congenitally missing teeth, and no previous orthodontic treatment. Before enrollment, participants and their parents or legal guardians (for minors) received detailed information regarding the study procedures and its anonymous nature. Written informed consent was obtained from all participants and from the parents or legal guardians of participants under 18 years of age. The study was approved by the Ethics Committee of the “Gaetano Martino” University Hospital, University of Messina (Approval No. 705). Participants were consecutively recruited over a one-year period, from April 2023 to April 2024. A total of 136 patients were assessed for eligibility; all met the eligibility criteria and agreed to participate. Thus, no patients were excluded or declined participation, and no participants withdrew after enrollment. The present study represents an expanded analysis of the recruitment population previously examined in our preliminary exploratory study [10]. Specifically, 126 participants were included in the previous study, whereas the present study included 10 additional participants, resulting in a total sample of 136. The current investigation addressed distinct research questions and extended the previous study by examining oral health-related quality of life using the OHIP-14 and its relationships with body image concerns and occlusal status through a distinct analytical framework. The final sample consisted of 136 adolescents aged 10 to 19 years (M = 15.49; SD = 2.64; 47.8% males and 52.2% females). According to Angle’s classification, 49 participants (36.0%) were classified as Class I, 45 (33.1%) as Class II, and 42 (30.9%) as Class III. All participants completed the assessment protocol in its entirety.

### 2.2. Instruments

The assessment protocol included a sociodemographic questionnaire collecting age and sex, clinical information on occlusal status according to Angle’s classification obtained during the orthodontic diagnostic assessment, and two validated self-report instruments assessing psychological and oral health-related outcomes, as described below.

#### 2.2.1. Body Image Concern Inventory (I-BICI)

The Body Image Concern Inventory (BICI) was originally developed and validated by Littleton and colleagues [43,44] to assess appearance-related concerns, including body image dissatisfaction, checking and camouflaging behaviors, and social avoidance. The Italian version of the instrument was validated by Luca et al. [45], demonstrating high sensitivity (96%) and specificity (76%).

The instrument comprises 19 items organized into a two-factor structure, with responses recorded on a 5-point Likert scale. The first factor, Dysmorphic Symptoms, consists of 12 items assessing shame, dissatisfaction, and distress related to physical appearance. The second factor, Symptom Interference, comprises 7 items evaluating the impact of body image concerns on psychosocial functioning and activities of daily living. Items are scored from 1 (never) to 5 (always), with higher scores indicating greater body image concerns. No items are reverse-coded. Item scores are summed to obtain the BICI Total score, with a theoretical range of 19–95. Based on the 12-item Dysmorphic Symptoms and 7-item Symptom Interference subscales, the corresponding theoretical ranges are 12–60 and 7–35, respectively. As all participants completed the assessment protocol in its entirety, no missing-item imputation or prorating procedures were required. The Italian validation study reported excellent internal consistency, with Cronbach’s alpha coefficients of 0.93 for the total scale, 0.92 for the Dysmorphic Symptoms subscale, and 0.76 for the Symptom Interference subscale [45]. In the present sample, the BICI demonstrated excellent internal consistency for the total score (Cronbach’s α = 0.939) and the Dysmorphic Symptoms subscale (Cronbach’s α = 0.936), whereas the Symptom Interference subscale showed good internal consistency (Cronbach’s α = 0.814).

#### 2.2.2. Oral Health Impact Profile (OHIP-14)

The Oral Health Impact Profile-14 (OHIP-14) is a self-report instrument comprising 14 items distributed across seven domains that assess the impact of oral health conditions on daily functioning and well-being, including functional limitation, physical pain, psychological discomfort, physical disability, psychological disability, social disability, and handicap [46]. The items assess subjective perceptions related to oral health and are rated on a 5-point Likert scale.

The OHIP-14 can be scored using two different methods. The first, known as the additive method, consists of summing the scores of all items to obtain a total score ranging from 0 to 56. The second, referred to as the simple count method, calculates the number of items endorsed with responses ranging from “sometimes” to “very often,” yielding scores between 0 and 14. In both scoring procedures, higher scores indicate poorer perceived oral health and a greater psychosocial impact of oral conditions. In the present study, the additive scoring method was used for all analyses. Responses to each item were scored from 0 (never) to 4 (very often), with no reverse-coded items. The 14-item scores were summed to obtain an OHIP-14 Total score ranging from 0 to 56, with higher scores indicating poorer oral health-related quality of life. Each of the seven domains comprises two items; domain scores were calculated by summing the corresponding item scores and therefore ranged from 0 to 8. As all participants completed the assessment protocol in its entirety, no missing-item imputation or prorating procedures were required. The Italian validation of the OHIP-14 demonstrated excellent internal consistency, with a Cronbach’s alpha coefficient of 0.90 [46]. In the present sample, the OHIP-14 Total score demonstrated excellent internal consistency (Cronbach’s α = 0.942). Cronbach’s alpha coefficients for the individual domains were 0.576 for Functional limitation, 0.787 for Physical pain, 0.725 for Psychological discomfort, 0.725 for Physical disability, 0.680 for Psychological disability, 0.834 for Social disability, and 0.830 for Handicap.

### 2.3. Statistical Analysis

Data are presented as means and standard deviations for continuous variables and as frequencies and percentages for categorical variables. The normality of continuous variables was assessed using the Kolmogorov–Smirnov test. As several variables did not meet the assumption of normality, non-parametric analyses were performed where appropriate. The internal consistency of the study measures was assessed using Cronbach’s alpha coefficients. Spearman’s rank correlation coefficient was used to examine associations between body image concerns, as assessed by the BICI, and oral health-related quality of life, as assessed by the OHIP-14. The Mann–Whitney U test was used to examine differences in BICI and OHIP-14 scores between male and female participants. For Hypotheses 1 and 2, analyses involving the BICI Total and OHIP-14 Total scores constituted the primary inferential focus, whereas analyses involving BICI subscales and individual OHIP-14 domains were considered secondary and exploratory and were not adjusted for multiple comparisons. Generalized linear models were subsequently used to examine the adjusted associations of age, sex, and Angle class with BICI Total and OHIP-14 Total scores. For both outcomes, the primary models specified a Gaussian distribution with an identity link function and included age as a continuous covariate and sex and Angle class as categorical factors. Angle Class III and male sex were used as the reference categories. The scale parameter was estimated by maximum likelihood, and model-based covariance estimates were used. Results are reported as unstandardized regression coefficients (B) with 95% confidence intervals. Overall model fit was assessed using the likelihood-ratio omnibus test and goodness-of-fit statistics. Model adequacy was further evaluated through examination of standardized Pearson residuals, including their distribution, normal Q-Q plots, and plots of standardized Pearson residuals against predicted values. Residual diagnostics were satisfactory for the BICI Total model. For the OHIP-14 Total model, residual diagnostics indicated right-skewness and non-constant residual variance. Therefore, a sensitivity analysis using a Tweedie distribution with a log link function was conducted to assess the robustness of the adjusted associations to an alternative distributional specification. As an additional sensitivity analysis, age-by-Angle class interaction terms were included in separate models for BICI Total and OHIP-14 Total scores to examine whether the associations between Angle class and the two outcomes varied as a function of age. Statistical analyses were performed using SPSS Statistics version 25 for Windows. A *p*-value < 0.05 was considered statistically significant.

## 3. Results

Descriptive statistics of the study variables are presented in Table 1.

### 3.1. Hypothesis 1

Spearman’s correlation coefficients are presented in Table 2. BICI Total score was positively associated with OHIP-14 Total score (ρ = 0.495, *p* < 0.001), supporting the hypothesized association between body image concerns and oral health-related quality of life. Secondary exploratory analyses at the domain level showed positive associations between BICI measures and individual OHIP-14 domains. In particular, BICI Total score showed moderate positive correlations with Psychological discomfort (ρ = 0.482), Psychological disability (ρ = 0.514), and Physical disability (ρ = 0.427). Similar patterns were observed for BICI Dysmorphic Symptoms and BICI Symptom Interference. Given the number of domain-level comparisons, these exploratory findings were interpreted primarily in terms of the magnitude and direction of the associations rather than statistical significance alone.

Overall, the association between BICI Total and OHIP-14 Total scores supported Hypothesis 1, indicating that greater body image concerns were associated with poorer oral health-related quality of life.

### 3.2. Hypothesis 2

Results of the Mann–Whitney U tests are presented in Table 3. Female participants reported higher BICI Total scores than male participants (41.00 ± 16.92 vs. 32.25 ± 17.11, *p* = 0.003, *r* = 0.252), indicating greater body image concerns, whereas OHIP-14 Total scores were similar between female and male participants (13.30 ± 13.60 vs. 11.92 ± 12.32, *p* = 0.688, *r* = 0.034). Secondary exploratory analyses showed higher BICI Dysmorphic Symptoms scores among female participants (36.01 ± 14.53 vs. 27.85 ± 14.54, *p* = 0.001, *r* = 0.272), whereas the effect size for BICI Symptom Interference was small (*r* = 0.055). Exploratory analyses of the individual OHIP-14 domains likewise showed small effect sizes (*r* = 0.003–0.132). Given the number of secondary comparisons, these exploratory findings were interpreted primarily in terms of the magnitude and direction of the observed differences rather than statistical significance alone.

Overall, these findings partially support Hypothesis 2, with sex differences observed for body image concerns but not for oral health-related quality of life at the total-score level.

### 3.3. Hypothesis 3

Mean BICI Total scores were 23.94 (SD = 11.57), 40.56 (SD = 16.86), and 47.83 (SD = 14.48) for Angle Classes I, II, and III, respectively. Mean OHIP-14 Total scores were 6.37 (SD = 5.26), 17.80 (SD = 17.12), and 14.43 (SD = 11.31), respectively. Standardized mean differences indicated a large difference in BICI Total scores between Angle Classes I and III (Cohen’s *d* = −1.84) and a smaller difference between Classes II and III (*d* = −0.46). For OHIP-14 Total scores, the difference between Classes I and III was also large (*d* = −0.94), whereas the difference between Classes II and III was small (*d* = 0.23).

Results of the generalized linear models are presented in Table 4. In models including age, sex, and Angle class, age was not significantly associated with either BICI Total score (*p* = 0.700) or OHIP-14 Total score (*p* = 0.538). Sex was independently associated with BICI Total score, with males reporting significantly lower levels of body image concerns than females (B = −5.69, 95% CI = −10.59 to −0.80, *p* = 0.023), whereas no significant association was observed for OHIP-14 Total score (*p* = 0.928). Compared with participants with Angle Class III malocclusion, those with Angle Class I exhibited significantly lower BICI Total scores (B = −22.65, 95% CI = −28.26 to −17.03, *p* < 0.001) and OHIP-14 Total scores (B = −8.06, 95% CI = −11.90 to −4.22, *p* < 0.001). In addition, participants with Angle Class II showed significantly lower BICI Total scores than those with Angle Class III (B = −6.53, 95% CI = −13.02 to −0.03, *p* = 0.049), whereas no significant difference was found for OHIP-14 Total score (*p* = 0.270). In an additional sensitivity analysis, the age-by-Angle class interaction was not significant for either BICI Total (Wald χ^2^(2) = 0.40, *p* = 0.818) or OHIP-14 Total (Wald χ^2^(2) = 1.42, *p* = 0.492), providing no evidence that the associations between Angle class and either outcome varied significantly as a function of age within the age range examined. The sensitivity analysis using a Tweedie distribution with a log link function confirmed the pattern observed in the primary analysis for the OHIP-14 Total score. Angle class remained significantly associated with OHIP-14 Total score (Wald χ^2^(2) = 30.92, *p* < 0.001), whereas neither age (Wald χ^2^(1) = 0.27, *p* = 0.603) nor sex (Wald χ^2^(1) = 0.001, *p* = 0.982) was significantly associated with the outcome. The contrast between Angle Classes I and III remained significant (*p* < 0.001), whereas the contrast between Angle Classes II and III remained non-significant (*p* = 0.235).

## 4. Discussion

The present study investigated the associations among occlusal status, body image concerns, and oral health-related quality of life in adolescents with malocclusion. Overall, the findings provided substantial support for the study hypotheses, showing significant associations between body image concerns and oral health-related quality of life. Furthermore, significant sex differences emerged in body image concerns, whereas no differences were observed in oral health-related quality of life. Finally, generalized linear models showed that Angle class was independently associated with both body image concerns and oral health-related quality of life after controlling for age and sex, while sex was independently associated with body image concerns only. The following sections discuss these findings in relation to the existing literature.

The findings related to Hypothesis 1 supported an association between body image concerns and oral health-related quality of life, with greater BICI Total scores associated with poorer OHIP-14 Total scores. Secondary exploratory analyses showed a consistent pattern of positive associations involving BICI subscales and individual OHIP-14 domains, although these domain-level findings should be interpreted cautiously given the number of comparisons. The interpretation of these exploratory domain-level findings should also consider the variable internal consistency observed across the OHIP-14 domains, particularly the lower coefficients for Functional Limitation and Psychological Disability, which may limit the reliability of findings involving these domains. These findings are in agreement with previous evidence suggesting that dissatisfaction with dental appearance and self-perceived oral health are closely related to body image dissatisfaction and psychosocial well-being in young individuals [47]. More broadly, research among adolescent orthodontic patients has emphasized the relevance of individual and psychosocial factors, including dental appearance-related perceptions, in understanding oral health-related quality of life [48], while evidence syntheses have further highlighted the multifactorial nature of oral health-related quality of life during adolescence [49].

The findings related to Hypothesis 2 partially supported the proposed hypothesis. Female participants reported greater body image concerns than males at the BICI Total score level, whereas OHIP-14 Total scores were similar between sexes. Secondary exploratory analyses showed higher Dysmorphic Symptoms scores among female participants, while differences in Symptom Interference and individual OHIP-14 domains were small. These findings suggest that sex differences in adolescents with malocclusion primarily concerned appearance-related perceptions rather than oral health-related quality of life.

The greater body image concerns observed among female participants are consistent with previous evidence indicating that adolescent girls generally report higher levels of appearance-related dissatisfaction and greater psychological sensitivity to perceived physical imperfections than boys [50]. Within the orthodontic literature, Iranzo-Cortés et al. [26] identified female sex as one of the factors associated with a greater psychological impact of malocclusion in adolescents. Similarly, Džemidžić et al. [51] reported that the overall psychosocial impact of malocclusion was broadly comparable between boys and girls, although female participants expressed greater concern regarding dental aesthetics. Together, these findings suggest that female adolescents may be particularly sensitive to the aesthetic implications of malocclusion, even when the overall psychosocial burden does not substantially differ between sexes.

Conversely, the small effect observed for the OHIP-14 Total score suggests that male and female adolescents reported broadly comparable levels of oral health-related quality of life. Although females reported greater concerns regarding their physical appearance, exploratory analyses of the individual OHIP-14 domains likewise indicated only small sex differences and should be interpreted cautiously given the number of comparisons. These findings are consistent with previous evidence indicating that the psychological experience of malocclusion is related to individuals’ subjective perception of dental and facial aesthetics, whereas sex alone does not necessarily determine oral health-related quality of life [26,52].

The findings related to Hypothesis 3 largely supported the proposed hypothesis, demonstrating that Angle class was independently associated with both body image concerns and oral health-related quality of life after controlling for age and sex. In contrast, age was not independently associated with either outcome, whereas sex was independently associated with body image concerns only. These findings suggest that the clinical pattern of malocclusion according to Angle’s classification [42] may represent a relevant correlate of adolescents’ body image concerns and oral health-related quality of life, independently of age and sex.

A particularly noteworthy finding was that adolescents with Angle Class III malocclusion reported greater body image concerns than those with Angle Class I, whereas the difference between Angle Classes III and II was smaller and should be interpreted cautiously given the confidence interval approaching the null value. This finding indicates that body image concerns differed across Angle classes, with adolescents classified as Angle Class III reporting the highest levels among the groups examined. These findings are consistent with previous evidence indicating that the psychological impact of malocclusion is influenced by clinical characteristics of the condition and by adolescents’ subjective perception of facial appearance [26].

The generalized linear models further demonstrated that Angle Class III was associated with poorer oral health-related quality of life compared with Angle Class I, whereas no significant difference emerged between Angle Class II and Angle Class III after adjustment for age and sex. These findings indicate that oral health-related quality of life differed between specific Angle classes, with participants classified as Angle Class III reporting poorer outcomes than those classified as Angle Class I.

Beyond statistical significance, the magnitude and potential clinical relevance of these between-class differences should also be considered. Standardized mean differences indicated large differences between Angle Classes I and III for both BICI Total and OHIP-14 Total scores, whereas differences between Classes II and III were smaller. The clinical interpretation of these differences, however, requires caution. For the OHIP-14, minimally important differences reported in previous studies have varied across populations and clinical contexts. A 5-point minimally important difference has been reported in a non-orthodontic clinical population [53], whereas a prospective study of adults undergoing fixed orthodontic treatment estimated a minimally important difference of at least 15 points [54]. These benchmarks are not directly transferable to the present cross-sectional adolescent sample. Accordingly, although the adjusted 8.06-point difference in OHIP-14 Total score between Angle Classes I and III indicates a substantial between-group difference, it should not be interpreted as establishing clinical significance according to a validated threshold for this population. Similarly, although the BICI differences were large in standardized terms, in the absence of a validated minimally important difference applicable to the present population, these findings cannot establish clinically significant body image disturbance.

With regard to the other variables included in the models, sex was independently associated with body image concerns but not oral health-related quality of life, whereas age was not associated with either outcome. These findings suggest that body image concerns are associated with both clinical characteristics and sex, whereas oral health-related quality of life may also vary according to the clinical pattern of malocclusion, as defined by Angle’s classification. Taken together, the present findings indicate that Angle class was independently associated with both body image concerns and oral health-related quality of life, while greater body image concerns were associated with poorer oral health-related quality of life at the total-score level. Furthermore, female adolescents reported greater appearance-related concerns. Collectively, these findings are consistent with a biopsychosocial perspective in which clinical characteristics and subjective experiences are considered jointly when evaluating adolescents with malocclusion. From a clinical perspective, these findings highlight the importance of complementing conventional orthodontic assessment with the evaluation of body image concerns and patient-reported oral health-related quality of life. The routine assessment of body image concerns and oral health-related quality of life may help identify adolescents reporting greater appearance-related concerns or poorer perceived oral health-related quality of life despite comparable clinical conditions. In particular, adolescents with Angle Class III malocclusion who report substantial body image concerns or poorer oral health-related quality of life may warrant further psychological assessment when clinically indicated, within a multidisciplinary approach to care. Such an approach may contribute to a more comprehensive understanding of patients’ needs and ultimately promote more patient-centered orthodontic care. However, the applicability of this multidisciplinary perspective across different cultural and healthcare contexts should be evaluated in future studies rather than assumed from the present single-center findings.

## 5. Strengths and Limitations

Several strengths of this study deserve consideration. To our knowledge, this is among the first investigations to simultaneously examine the relationships among malocclusion, body image concerns, and oral health-related quality of life in an adolescent population within a comprehensive biopsychosocial framework. Furthermore, the use of validated psychometric instruments, including the BICI and the OHIP-14, enabled the assessment of complementary dimensions that have rarely been investigated together in orthodontic research. An additional methodological strength is represented by the application of generalized linear models, which allowed the independent associations of Angle class, age, and sex with the study outcomes to be examined while controlling for the other variables included in the models.

Nevertheless, several limitations should be acknowledged. First, the cross-sectional design precludes causal inferences regarding the relationships among malocclusion, body image concerns, and oral health-related quality of life. Moreover, because all participants had a diagnosis of malocclusion and no normal-occlusion comparison group was included, the present findings cannot establish whether adolescents with malocclusion differ from unaffected peers in body image concerns or oral health-related quality of life.

Second, participants were recruited from a single Italian university hospital, potentially limiting the generalizability of the findings to adolescents from other geographic, cultural, and healthcare contexts. Furthermore, because participants were recruited among adolescents already attending an orthodontic clinic, the sample may have been selectively enriched for individuals with greater treatment motivation or heightened concerns about dental and facial appearance. This potential selection bias should be considered when generalizing the findings to adolescents with malocclusion in the general population. Cultural perceptions of dental and facial appearance, access to orthodontic care, socioeconomic circumstances, and the distribution of malocclusion phenotypes may differ across populations and could influence body image concerns and oral health-related quality of life. Accordingly, the pattern of associations observed across Angle classes should not be assumed to generalize uniformly to other populations. In addition, Angle classification was independently performed by two orthodontists, with any disagreements resolved through discussion and consensus. Although no formal pre-study calibration or quantitative assessment of inter-examiner agreement was performed, independent assessment followed by consensus was used to establish the final clinical classification.

Third, analyses involving BICI subscales and individual OHIP-14 domains were exploratory and were not adjusted for multiple comparisons. Moreover, internal consistency varied across the OHIP-14 domains, with lower coefficients observed for Functional Limitation and Psychological Disability. Therefore, domain-level findings should be interpreted cautiously and require confirmation in future studies. Fourth, psychological and patient-reported outcomes were assessed through self-report measures and may therefore be susceptible to reporting and social desirability biases.

Finally, although the sample included adolescents across the three Angle classes, potentially relevant sociodemographic and psychosocial variables, including socioeconomic status, family functioning, and psychiatric history, were not assessed. Their potential contribution to the observed associations could therefore not be examined, further limiting the extent to which the findings can be generalized across diverse populations.

Future longitudinal, multicenter studies involving larger and more geographically, culturally, and sociodemographically diverse samples are needed to clarify the temporal relationships among malocclusion, body image concerns, and oral health-related quality of life, to examine the reproducibility of the observed associations across populations and healthcare settings, to confirm the exploratory domain-level findings, and to identify additional clinical and psychosocial factors associated with these outcomes.

## 6. Conclusions

The findings of this study provide further evidence that malocclusion should be considered not only in terms of its structural and functional characteristics but also in relation to its psychosocial implications during adolescence. Angle class was significantly associated with body image concerns and oral health-related quality of life, while body image concerns demonstrated particularly strong associations with oral health-related quality of life. Moreover, Angle class remained independently associated with both outcomes after controlling for age and sex, highlighting the importance of considering body image concerns and oral health-related quality of life in clinical practice.

These findings support the adoption of a biopsychosocial approach to the assessment and management of adolescents with malocclusion. Integrating conventional orthodontic evaluation with the assessment of body image concerns and oral health-related quality of life may help identify adolescents reporting greater appearance-related concerns or poorer oral health-related quality of life and contribute to more individualized, patient-centered treatment planning. Further longitudinal and multicenter studies are needed to clarify the temporal and potentially causal pathways linking malocclusion, body image concerns, and oral health-related quality of life, and to further examine the clinical relevance of integrating these patient-reported outcomes into orthodontic assessment and care.

## Figures and Tables

**Table 1:** Descriptive statistics.

	Mean	SD
Age	15.49	2.64
BICI Total score	36.81	17.50
BICI Dysmorphic symptoms	32.11	15.04
BICI Symptom interference	4.70	3.28
OHIP-14 Total score	12.63	12.97
OHIP-14 Functional limitation	1.27	1.75
OHIP-14 Physical pain	3.14	2.39
OHIP-14 Psychological discomfort	2.34	2.39
OHIP-14 Physical disability	1.37	2.03
OHIP-14 Psychological disability	2.04	2.31
OHIP-14 Social disability	1.16	2.13
OHIP-14 Handicap	1.30	2.19

**Table 2:** Spearman’s correlation coefficients between body image concerns and oral health-related quality of life.

	BICI Total Score	BICI Dysmorphic Symptoms	BICI Symptom Interference
OHIP-14 Total score	0.495 **	0.475 **	0.449 **
OHIP-14 Functional limitation	0.310 **	0.287 **	0.309 **
OHIP-14 Physical pain	0.326 **	0.298 **	0.339 **
OHIP-14 Psychological discomfort	0.482 **	0.482 **	0.374 **
OHIP-14 Physical disability	0.427 **	0.389 **	0.460 **
OHIP-14 Psychological disability	0.514 **	0.512 **	0.420 **
OHIP-14 Social disability	0.354 **	0.329 **	0.402 **
OHIP-14 Handicap	0.389 **	0.359 **	0.421 **

Note. ρ = Spearman’s rank correlation coefficient. Analyses involving BICI subscales and individual OHIP-14 domains were considered secondary and exploratory and were not adjusted for multiple comparisons. ** *p* < 0.001.

**Table 3:** Sex differences in body image concerns and oral health-related quality of life.

	Males		Females				
	(M ± SD)	Median (Q1–Q3)	(M ± SD)	Median (Q1–Q3)	U	*p*-Value	Effect Size (*r*)
BICI Total score	32.25 ± 17.11	30.00 (22.00–44.50)	41.00 ± 16.92	41.00 (29.00–53.00)	1634.0	0.003 *	0.252
BICI Dysmorphic symptoms	27.85 ± 14.54	25.50 (19.00–37.75)	36.01 ± 14.53	37.00 (25.00–46.00)	1579.0	0.001 *	0.272
BICI Symptom interference	4.40 ± 3.13	4.00 (4.00–6.00)	4.99 ± 3.42	4.00 (4.00–7.00)	2167.0	0.521	0.055
OHIP-14 Total score	11.92 ± 12.32	8.50 (4.00–15.00)	13.30 ± 13.60	9.00 (5.00–16.00)	2215.5	0.688	0.034
OHIP-14 Functional limitation	1.25 ± 1.80	1.00 (0.00–2.00)	1.31 ± 1.73	1.00 (0.00–2.00)	2236.0	0.740	0.028
OHIP-14 Physical pain	3.22 ± 2.36	3.00 (1.00–5.00)	3.08 ± 2.45	3.00 (1.00–4.00)	2216.5	0.688	0.034
OHIP-14 Psychological discomfort	2.00 ± 2.21	1.00 (0.00–3.00)	2.66 ± 2.52	2.00 (0.00–4.00)	1931.5	0.126	0.132
OHIP-14 Physical disability	1.42 ± 2.02	0.50 (0.00–2.00)	1.34 ± 2.06	0.00 (0.00–2.00)	2203.5	0.621	0.042
OHIP-14 Psychological disability	1.77 ± 2.10	1.00 (0.00–3.00)	2.30 ± 2.49	1.00 (0.00–3.00)	2061.0	0.270	0.095
OHIP-14 Social disability	1.05 ± 1.95	0.00 (0.00–1.00)	1.28 ± 2.30	0.00 (0.00–1.00)	2300.0	0.970	0.003
OHIP-14 Handicap	1.28 ± 2.04	0.00 (0.00–2.00)	1.32 ± 2.35	0.00 (0.00–2.00)	2200.5	0.601	0.045

Note. Q1 = first quartile; Q3 = third quartile; U = Mann–Whitney U statistic; *r* = effect size calculated as |*Z*|/√N. Analyses involving BICI subscales and individual OHIP-14 domains were considered secondary and exploratory and were not adjusted for multiple comparisons. * Significant *p*-values (*p* < 0.05).

**Table 4:** Generalized linear models for body image concerns and oral health-related quality of life.

	Age		Sex		Angle Class (Class I vs. Class III)		Angle Class (Class II vs. Class III)	
	B (95% CI)	*p*	B (95% CI)	*p*	B (95% CI)	*p*	B (95% CI)	*p*
BICI Total score	0.17 (−0.69 to 1.02)	0.700	−5.69 (−10.59 to −0.80)	0.023 *	−22.65 (−28.26 to −17.03)	<0.001 *	−6.53 (−13.02 to −0.03)	0.049 *
OHIP-14 Total score	−0.25 (−1.06 to 0.55)	0.538	−0.20 (−4.44 to 4.05)	0.928	−8.06 (−11.90 to −4.22)	<0.001 *	3.39 (−2.63 to 9.42)	0.270

Note. B = unstandardized regression coefficient; CI = 95% confidence interval. Angle Class III and male sex were used as reference categories. * Significant *p*-values (*p* < 0.05).

## Data Availability

The data supporting the findings of this study are available from the corresponding author upon reasonable request.

## Abbreviations

The following abbreviations are used in this manuscript:

BICI	Body Image Concern Inventory
I-BICI	Italian Body Image Concern Inventory
OHIP-14	Oral Health Impact Profile-14
